# Isolated Foot Drop Due to a Cerebral Infarction Mimicking Lumbar Radiculopathy: A Case Report and Literature Review

**DOI:** 10.7759/cureus.76894

**Published:** 2025-01-04

**Authors:** Abdulkarim A Almutairi, Mishari S Alqahtani, Mohammed A Alsayari, Aser F Alamri

**Affiliations:** 1 Neuroscience Department, Neurology Division, Prince Sultan Military Medical City, Riyadh, SAU; 2 College of Medicine, King Saud Bin Abdulaziz University for Health Sciences, Riyadh, SAU

**Keywords:** acute cerebral infarction, drop-foot, ischemic cerebrovascular disease, pure motor monoparesis, stroke

## Abstract

Isolated foot drop is a neurological sign frequently linked to lower motor neuron (LMN) lesions, including peroneal nerve damage or L4-L5 radiculopathy. Nonetheless, upper motor neuron (UMN) lesions, such as strokes or tumors located in the parasagittal motor cortex, may sometimes manifest as isolated foot drops. The main causes of isolated foot drop secondary to central etiologies are uncommon, with few instances documented in the literature.

An 83-year-old male presented with a four-day history of left isolated foot drop that started in the big toe and then spread to involve the whole foot. Clinical examination was negative for any other neurological deficit. Magnetic resonance imaging (MRI) of the cervical, thoracic, and lumbar spine showed only mild lumbar spinal stenosis at the L4/L5 level. Brain MRI revealed acute infarction foci in the right superior frontal gyrus.

While uncommon, central causes of isolated foot drop should be taken into account when peripheral examinations yield inconclusive results. This case highlights the significance of a thorough diagnostic method, encompassing brain imaging, to detect lesions in the central nervous system. Timely identification and management of these cases are essential for enhancing patient outcomes and avoiding misdiagnosis.

## Introduction

Foot drop is a common neurological symptom characterized by weakness in the dorsiflexor muscles of the ankle and big toe. It is most often caused by lower motor neuron (LMN) lesions, such as damage to the peroneal nerve or a herniated disc at the L4-L5 level of the lumbar spine, leading to radiculopathy, which is a common cause of foot drop [1]. Rarely, foot drop can result from central lesions or upper motor neuron (UMN) lesions, such as a stroke or a mass affecting the parasagittal gyrus. Other causes of foot drop include trauma, muscular diseases, neuromuscular junction disorders, and spinal cord pathology. Therefore, a detailed history, complete neurological examination, and neurophysiological studies (i.e., electromyography, EMG/nerve conduction study, NCS) are crucial to differentiate between UMN and LMN lesions. An example of differentiating factors is that UMN lesions typically present with hyperreflexia, spasticity, and a positive Babinski sign, whereas LMN lesions are characterized by flaccid paralysis, muscle atrophy, and hyporeflexia. However, UMN lesions, such as those causing isolated foot drop, can mimic LMN conditions like peroneal neuropathy or L4/L5 radiculopathy. This overlap arises when the lesion is confined to specific motor pathways, resulting in isolated weakness without spasticity or hyperreflexia, creating a diagnostic challenge [2,3].

Central causes of isolated foot drop are uncommon, with fewer than 20 cases documented in the literature [2,4]. Among these, 10 cases have identified vascular etiologies as the underlying cause of isolated foot drop [3,5-13]. In this case report, we describe an 83-year-old male patient with right frontal parasagittal hypodensity, who presented with a sudden onset of isolated foot drop, mimicking peripheral peroneal neuropathy. The purpose of this article is to review the relevant literature on isolated central foot drop, highlight the diagnostic process, and explore subtle differences in the clinical presentation of our case compared to others reported in the literature, offering new insights into this extremely rare cause of isolated foot drop.

## Case presentation

An 83-year-old male presented to the Emergency Department (ED) complaining of left foot weakness. The weakness began four days ago, initially affecting the big toe. Over the subsequent four days, it gradually spread to involve the entire left foot by the time he presented to the ED. He did not complain of any other neurological symptoms, such as paresthesia or numbness. The patient denied any history of trauma to the legs or back, and there was no previous history of falls. His past medical history is positive for longstanding diabetes mellitus and hypertension. He has no previous history of cerebrovascular disease.

Upon examination, he was alert and oriented to time, place, and person. The cranial nerve examination was unremarkable. Muscle strength was normal in all limbs (5/5 on manual muscle testing (MMT)), except for the left foot, where dorsiflexion, plantarflexion, eversion, and inversion were completely weak (0/5 on MMT). Muscle tone and deep tendon reflexes (DTRs) were normal, with no pathological reflexes across all extremities. Sensory perception for pain, touch, vibration, and joint position was within normal limits. Distal pulses were intact in all extremities. Cerebellar examination showed no signs of ataxia or imbalance. His gait was impaired due to the weakness in his left foot. His initial National Institutes of Health Stroke Scale (NIHSS) score was 0.

**Figure 1:**
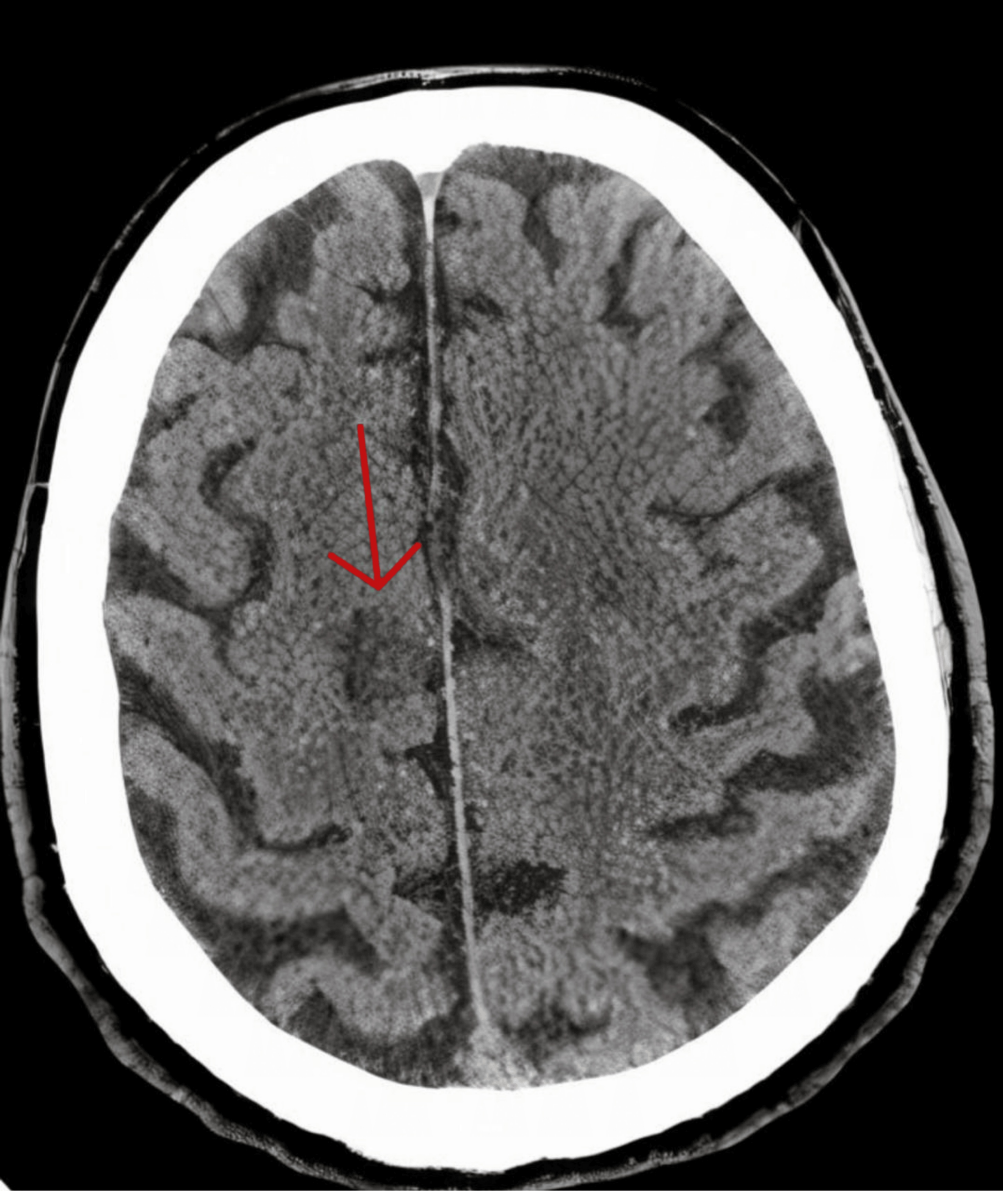
Axial non-contrast computed tomography of the brain Right frontal parasagittal hypodensity, likely vascular in nature, represents a subacute/chronic ischemic insult with chronic microangiopathic disease.

Laboratory tests, including complete blood count and biochemistry, were within normal limits, except for a creatinine level of 160 mmol/L (60-110 mmol/L) and blood urea nitrogen (BUN) of 10.9 mmol/L (3.6-7.1 mmol/L). His electrocardiogram (ECG) was normal, with sinus rhythm. Echocardiography showed asymptomatic degenerative severe aortic stenosis with mild left ventricular hypertrophy. Brain non-contrast computed tomography (NCCT) reported right frontal parasagittal hypodensity, likely vascular in nature, representing a subacute/chronic ischemic insult with chronic microangiopathic disease (Figure 1).

**Figure 2:**
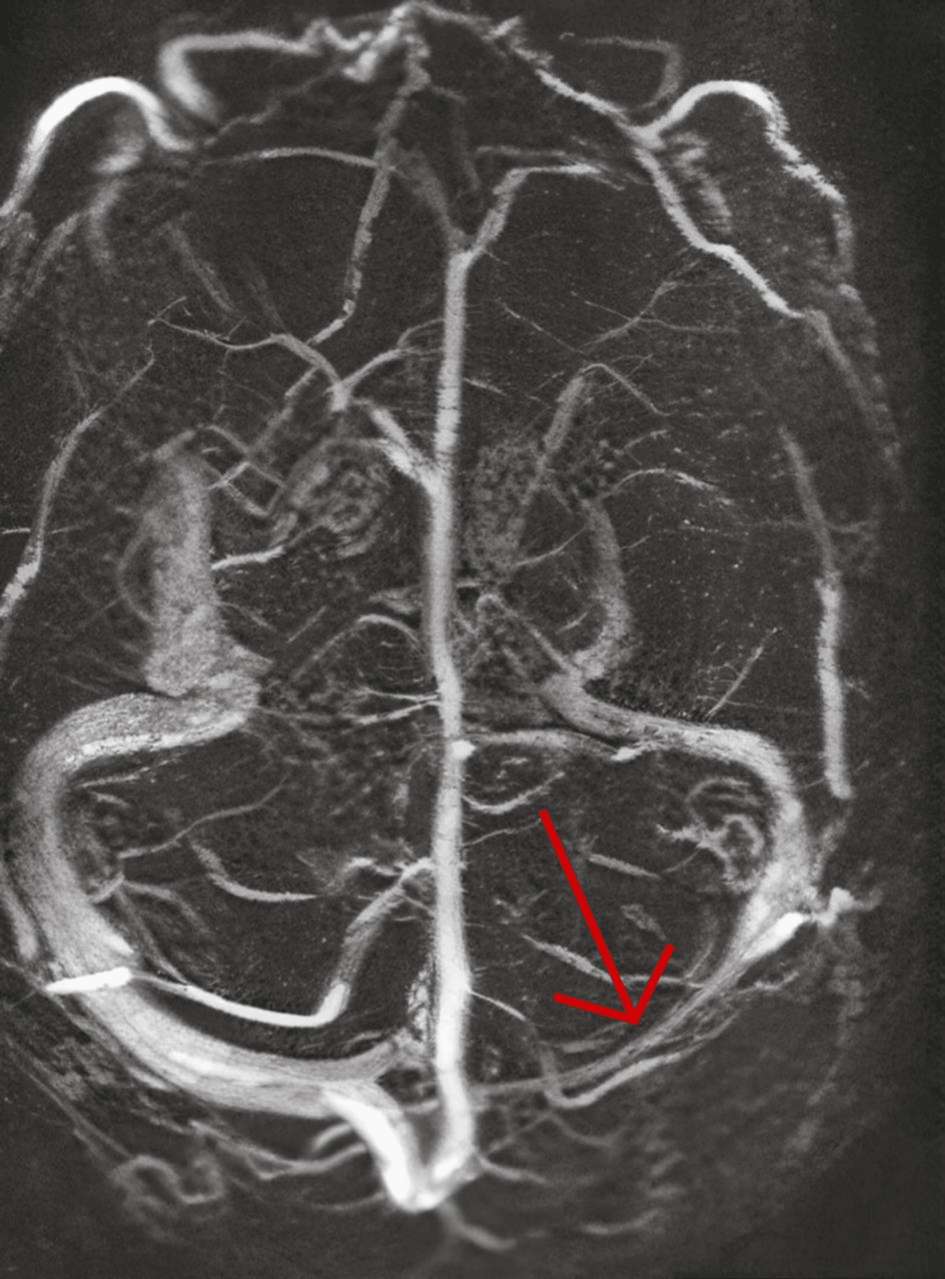
Magnetic resonance venography of the brain Relatively small left-sided sinuses, likely hypoplastic, show no evidence of significant cerebral venous thrombosis.

Brain magnetic resonance imaging/venography (MRI/MRV) without contrast was obtained. The MRV revealed relatively small left-sided sinuses, likely hypoplastic, with no evidence of significant cerebral venous thrombosis (Figure 2).

**Figure 3:**
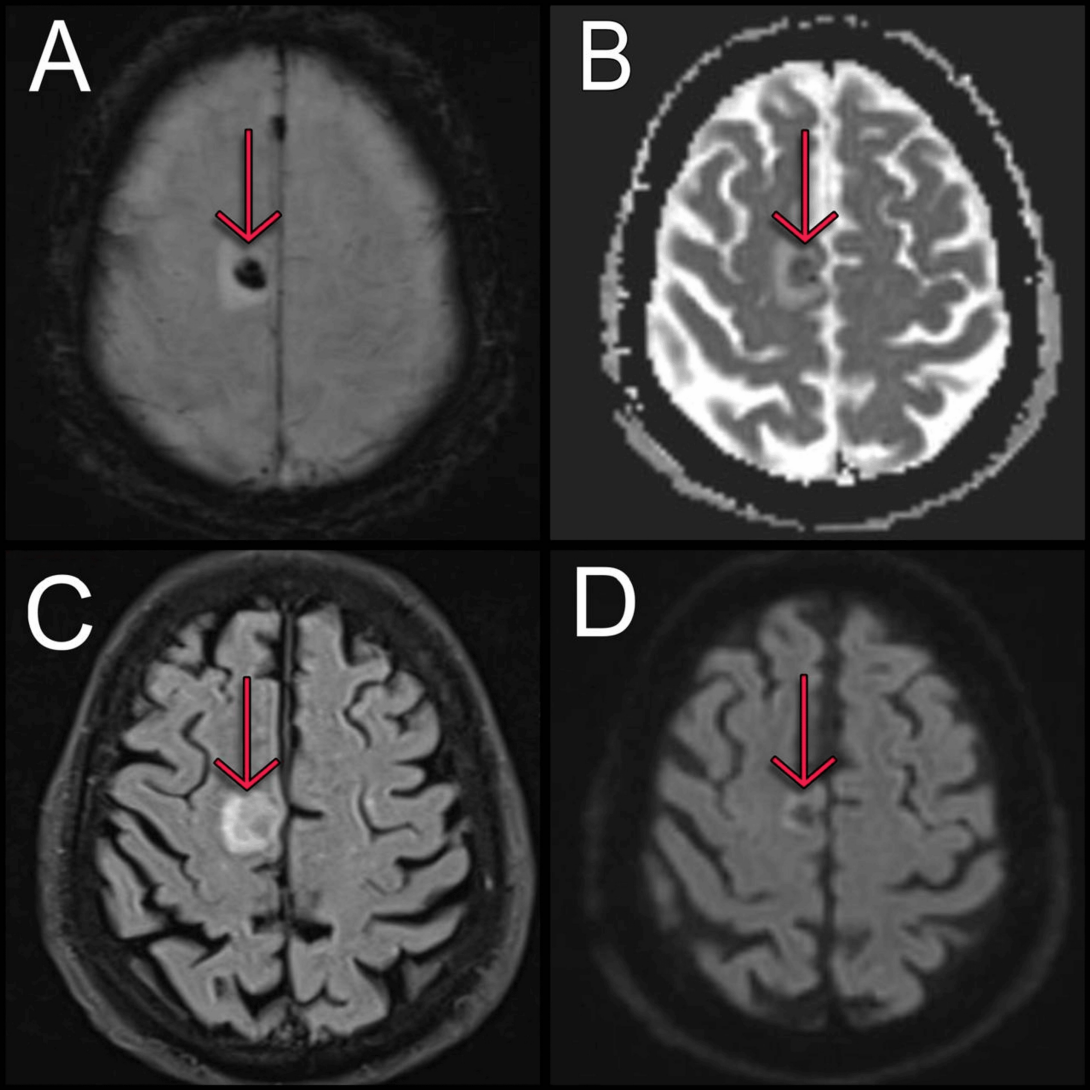
Magnetic resonance imaging sequences of the brain (A) Susceptibility-weighted imaging (SWI) of the brain showing right superior frontal gyrus involvement. (B) Apparent diffusion coefficient (ADC) showing a hypointense lesion in the right superior frontal gyrus. (C) Fluid-attenuated inversion recovery (FLAIR) reported a hyperintense lesion in the right superior frontal gyrus. (D) Diffusion-weighted imaging (DWI) revealing a hypointense lesion in the right superior frontal gyrus.

Conversely, MRI sequences demonstrated a cortically based process in the right superior frontal gyrus, with minimal mass effect and perilesional signal alteration, most likely vascular in origin due to its incomplete subcortical appearance (Figure 3).

**Figure 4:**
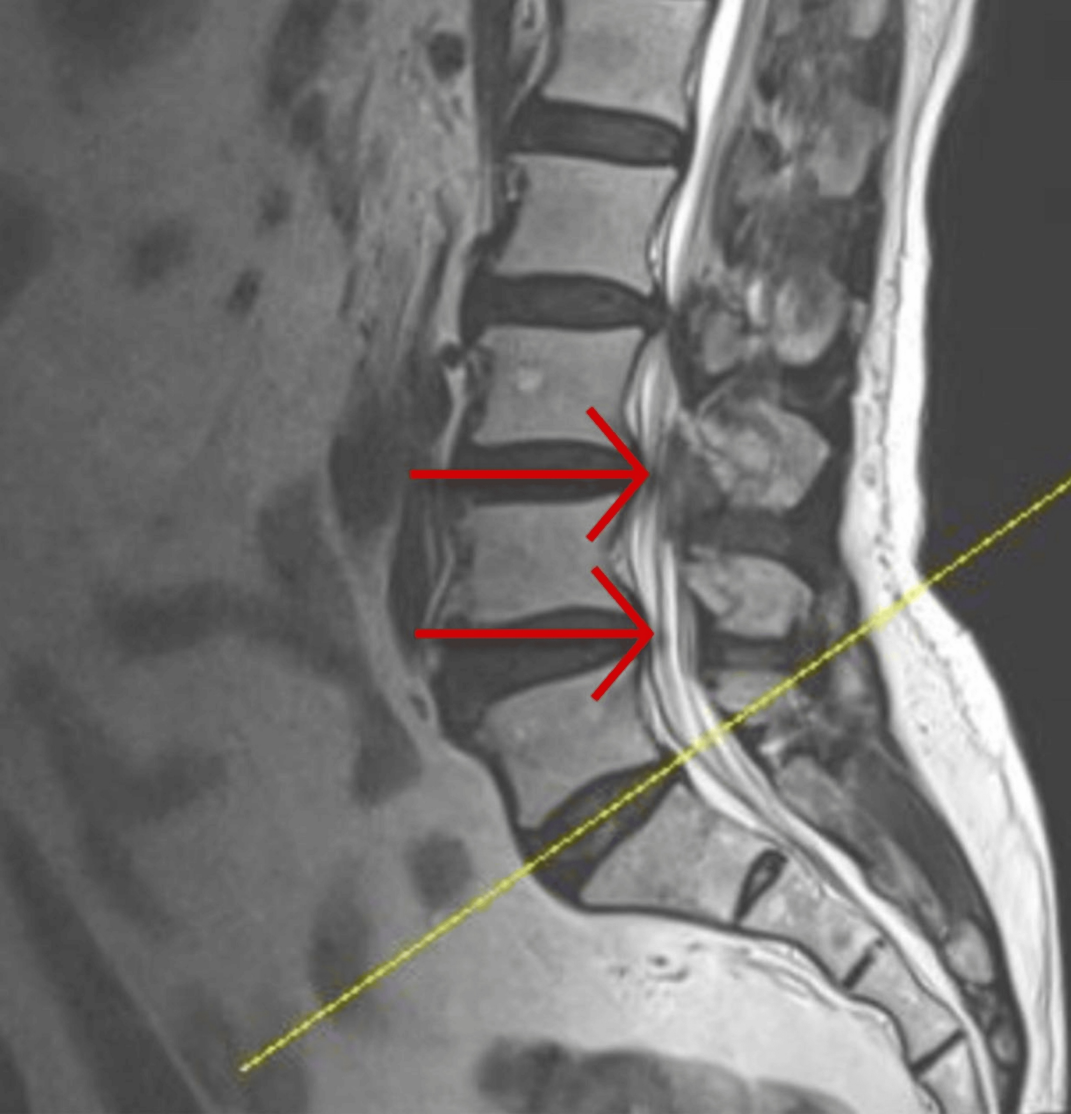
Magnetic resonance imaging of lumbar spine Asymmetric diffuse disc bulge at the L4/L5 level, resulting in mild spinal stenosis.

Transcranial Doppler (TCD) ultrasound was negative for any stenosis in the extracranial arteries. Lumbar spine MRI revealed multi-level diffuse degenerative changes and facet joint arthropathy. Additionally, there was an asymmetric diffuse disc bulge at the L4/L5 level, tilted to the left, resulting in mild spinal stenosis (Figure 4).

After five days of antiplatelet therapy and rehabilitation, the patient’s left leg muscle strength improved significantly from 0/5 to 4/5 on the MMT. Antiplatelet therapy enhanced blood flow to the affected brain region, preventing further ischemic damage, while rehabilitation focused on motor recovery, aiding in the recovery of muscle strength.

## Discussion

In the primary motor cortex, the dorsomedial surface of the cerebral hemispheres sends signals through the corticospinal tract to lower limb muscles, specifically the tibialis anterior, extensor hallucis longus, and extensor digitorum longus. These muscles control foot/ankle dorsiflexion and big toe extension, respectively. They are innervated primarily by the L4 and L5 nerve roots, with some contribution from the S1 root. Any disruption along this pathway can result in paresis, a condition characterized by weakened limb movement. Isolated foot drop is a weakness of these muscles without any other neurological deficit, such as pain. Peripheral neuropathy, such as peroneal nerve damage and L4/L5 radiculopathy, are common causes. However, central etiologies are rare, and there are multiple reports of isolated foot drop caused by cerebral insult, ranging from infarction to tumors, mimicking radiculopathy [2,3,5-13].

Diagnosing central causes of isolated foot drop requires a thorough neurological examination. Initial investigations, including spinal MRI and EMG/NCS, are essential to rule out peripheral causes. If these tests are negative and do not correlate with clinical symptoms, brain imaging (CT or MRI) should be performed to evaluate for central nervous system lesions. In our patient, brain MRI revealed a lesion in the right superior frontal gyrus, consistent with the clinical presentation, and involvement of the anterior cerebral artery (ACA) territory, suggesting a stroke mechanism.

Foot drop can result from cerebrovascular injury or tumor mass effect in the parasagittal motor cortex, the region responsible for ankle and toe movement. Table 1 summarizes 10 reported cases of acute cerebrovascular insult, with the majority resulting in unilateral foot drop (Table 1).

**Table 1:** Summary of published literature on acute foot drop from cerebral vascular etiologies M, male; F, female; MRI, magnetic resonance imaging; L, left; R, right; Y, years; DM, diabetes mellitus; HTN, hypertension; CKD, chronic kidney disease; APS, antiphospholipid syndrome; B/L, bilateral; HF, heart failure

Study	Vascular Etiology	Presenting Symptom	Age, Sex (M/F)	Area Affected	Comorbidities
Current case (2024)	Ischemic stroke	L, foot drop	83Y, M	Right superior frontal gyrus	DM, HTN
Tanıgör et al. (2019) [9]	Ischemic stroke	R, foot drop	60Y, F	Left precentral gyrus	DM, HTN, CKD, APS
Sweid et al. (2019) [6]	Ischemic stroke	B/L, foot drop	29Y, M	Multiple acute infarcts in both anterior cerebral hemispheres	Bipolar disorder
Kaykisiz and Unluer (2017) [3]	Ischemic stroke	L, foot drop	81Y, F	Right precentral gyrus	Medically free
Kim et al. (2015) [5]	Ischemic stroke	L, foot drop, paresthesia, and lower back pain	71Y, M	Right paracentral lobule and the parietal medial cortex	Lumbar radiculopathy in L4/5
Ricarte et al. (2014) [7]	Ischemic stroke	R, foot drop	43Y, M	Left frontal cortex	Medically free
Kim et al. (2014) [11]	Ischemic stroke	R, foot drop	74Y, M	Left precentral gyrus	Medically free
Park et al. (2013) [8]	Ischemic stroke	R, foot drop, vertigo	53Y, F	Right supplemental motor area	Hyperlipidemia
Oktem et al. (2012) [12]	Hemorrhagic contusion	L, foot drop	74Y, F	Right parasagittal region	HF, multitrauma
Teufack et al. (2011) [10]	Ischemic stroke	B/L, foot drop	56Y, M	B/L parasagittal frontal gyri	HTN, hepatitis C
Ku et al. (2007) [13]	Ischemic stroke	L, foot drop	68Y, M	Right precentral gyrus	HTN

Notably, Sweid et al. [6] described a 29-year-old male with acute bilateral foot drop, a history of bipolar disorder, and an anterior communicating artery (ACoA) aneurysm. Similarly, Teufack et al. [10] reported a 56-year-old male with hypertension who developed bilateral foot drop following ACoA treatment, with MRI revealing bilateral parasagittal frontal gyrus insult.

In our case, the patient presented with a left-sided foot drop, and an MRI confirmed a lesion in the right superior frontal gyrus, consistent with the clinical presentation of contralateral limb weakness. Park et al. [8] also reported a 53-year-old woman with sudden left foot drop preceded by vertigo, with functional MRI (fMRI) showing involvement of the contralateral medial precentral gyrus and supplementary motor area (SMA) of the paracentral lobule.

Central isolated foot drop caused by tumor mass effect is rare. Bilić et al. [2] highlighted that parasagittal meningiomas causing foot drop have only been documented in eight cases to date. Other rare central causes include cerebral metastases, demyelination plaques, and brain abscesses [4].

## Conclusions

Acute foot drop is often peripheral in origin but can also result from central causes, as shown in our case and supported by a review of prior reports. Most cases in the literature describe unilateral foot drop from cerebrovascular events, with rare bilateral presentations linked to specific vascular regions like ACoA aneurysms. Our patient’s left foot drop, caused by a right superior frontal gyrus lesion, highlights the need to consider contralateral central causes. Recognizing UMN signs, such as hyperreflexia, a positive Babinski sign, and intact peripheral sensation, can help identify central origins. Early detection is crucial, as these conditions may require urgent diagnosis and intervention. This review emphasizes the need for a comprehensive assessment of foot drop, including consideration of central causes, to enhance diagnostic precision, avoid misdiagnosis, and optimize patient outcomes.
